# Unlocking therapeutic potential: dual gene therapy for ameliorating the disease phenotypes in a mouse model of *RPE65* Leber congenital amaurosis

**DOI:** 10.3389/fmed.2023.1291795

**Published:** 2024-01-09

**Authors:** Yanbo Liu, Jingjie Tai, Chaofeng Yu, Dan Xu, Dan Xiao, Jijing Pang

**Affiliations:** ^1^Eye Institute of Xiamen University, Fujian Provincial Key Laboratory of Ophthalmology and Visual Science, School of Medicine, Xiamen University, Xiamen, China; ^2^Shenyang Weijing Biotechnology Co., Ltd., Shenyang, China; ^3^Xiamen University Affiliated Xiamen Eye Center, Xiamen, China; ^4^Shenyang He Eye Specialist Hospital, Shenyang, China; ^5^Institute of Innovation Research for Precision Medical Treatment, He University, Shenyang, China

**Keywords:** gene replacement therapy, anti-apoptosis therapy, *rd12* mice, *RPE65*, *Bcl-2L10*, AAV

## Abstract

Leber congenital amaurosis (LCA) is the most common genetic cause of congenital visual impairment in infants and children. Patients with LCA who harbor *RPE65* mutations exhibit a deficiency in photoreceptor rhodopsin, leading to severe night blindness and visual impairment following birth. Since either gene replacement therapy or anti-apoptosis therapy alone cannot maintain both functional and morphological normality for a long time in the animal model, we propose a robust treatment strategy, that is, gene replacement therapy combined with anti-apoptotic therapy to protect photoreceptors from further degeneration while compensating for lost *RPE65* function. Here, *rd12* mice were injected subretinally at postnatal day 14 with four vector administrations, respectively. At 6 months after treatment, it was discovered that injection of three vectors, AAV8 (Y733F)-CBA-hRPE65, AAV8(Y733F)-CBA-hRPE65-BCL-2-L10 and mixture of half-dose AAV8(Y733F)-CBA-hRPE65 and half-dose AAV8 (Y733F)-CBA-BCL-2-L10, could partially restore the visual function of *rd12* mice. Meanwhile, these treated eyes also exhibited a thicker outer nuclear layer (ONL) structure. However, despite the fact that the eyes of *rd12* mice injected with the AAV8 (Y733F)-CBA-BCL-2-L10 vector displayed a slightly thicker ONL structure compared to untreated eyes, the visual function of the treated eyes did not recover. Continuing the observation period to 12 months after treatment, we found that compared to *rd12* mice at 6-month post-treatment, *rd12* mice injected with AAV8 (Y733F)-CBA-hRPE65 or mixture of half-dose AAV8(Y733F)-CBA-hRPE65 and half-dose AAV8 (Y733F)-CBA-BCL-2-L10 exhibited varying degrees of decline in both visual function and ONL thickness. However, in the case of *rd12* mice injected with the AAV8(Y733F)-CBA-hRPE65-BCL-2-L10 vector, the ONL thickness remains consistent at both 6 and 12 months after treatment. These mice continued to maintain a relatively strong visual function and showed restoration in the levels of *RPE65* and Rhodopsin protein expression. Our findings illustrate that early postnatal treatment with AAV vectors containing both the *hRPE65* gene and the *Bcl-2L10* anti-apoptotic gene provide enhanced and sustained retinal protection.

## Introduction

Leber congenital amaurosis (LCA) represents the most severe form of inherited retinal dystrophies, accounting for 5% of all cases, and contributes to up to 20% of congenital blindness in children. LCA manifests as a constellation of symptoms, encompassing nystagmus, sluggish or absent pupillary responses, and a non-recordable electroretinogram (1). LCA is essentially an autosomal recessive disorder, although rare autosomal dominant inheritance has been noted. The disease is known to be linked to mutations in approximately 38 different genes, illustrating the immense genetic heterogeneity of this pathology (2, 3). The most extensively studied form is LCA2, resulting from mutations in the *RPE65* gene that leads to deficits in the visual cycle (4).

Traditional therapeutic modalities for LCA have revolved around symptomatic management and have included visual aids, mobility training, and educational support. The objective has been to help patients adapt to their visual impairment rather than addressing the underlying genetic defects. However, with the advent of personalized medicine, gene therapy has emerged on the horizon as a potent strategy for tackling inherited diseases like LCA (5). Currently, adeno-associated virus (AAV) stands as the forefront platform for delivering gene therapies *in vivo*, ensuring both safety and efficacy. A significant proportion of AAV gene therapy under clinical development is directed towards the central nervous system, including the brain (6, 7) and eye (8–10). The most successful exemplar to date is Luxturna, an AAV-*RPE65* gene therapy that was approved by the FDA in 2017 (11). By supplementing the defective gene, it brings about a significant improvement in the visual function of treated areas in patients with LCA2 (12). However, the treatment strategy lacks the ability to maintain long-term therapeutic effects (13, 14). This problem may arise due to the irreversible apoptosis processes that are already underway in visual cells before treatment (15). Therefore, there are still a lot of issues with gene therapy technologies to be solved, including the potential for immune responses, the efficient delivery of the therapeutic gene to retinal cells, and the durability of the treatment effect (16).

The Bcl-2 family plays vital roles in apoptotic regulation and was initially discovered in the cancer setting (17–19). The current study has shown that a Bax/Bcl-2 ratio determines the ultimate fate of the cell, survival, or death (20). In the degenerating retinas of *RPE65*-deficient mice, there is an observed increase in pro-apoptotic factor Bax and a decrease in anti-apoptotic factor Bcl-2 (21). Considering that a significant majority of LCA2 patients are in a middle-advanced stage, where their visual cells have initiated apoptosis before receiving treatment, approaches focusing on a single aspect—be in gene replacement or apoptosis inhibition-might not result in lasting and enduring benefits (22, 23). To accomplish the objective of decelerating or halting the apoptotic progression in degenerated photoreceptor cells and concurrently restoring the patient’s vision, we propose a dual-action strategy that combines gene augmentation with apoptosis inhibition. This strategy involves delivering the normal *RPE65* gene while simultaneously utilizing anti-apoptotic factors *Bcl-2L10*. In this study, we introduce innovative strategies aimed at advancing the treatment of advanced LCA2. Despite being in preliminary phases, we will undertake further investigation that could potentially enhance the long-term prognosis for individuals affected by LCA2.

## Materials and methods

### Mice

The *rd12* mice were purchased from the Jackson Laboratory (Jackson Laboratory; Bar Harbor). C57BL/6J mice were purchased from Xiamen University Laboratory Animal Center (Xiamen, China). The ratio of males to females was relatively equal in all experiments. All mice were bred and maintained in the animal facility of Xiamen University Laboratory Animal Center. All mice were maintained in a standard pathogen-free environment at a 12-h light–dark cycle (from 8:00 a.m. to 8:00 p.m.) and had free access to water and food. All experimental were conducted in accordance with the Association for Research in Vision and Ophthalmology’s Statement for the Use of Animals in Ophthalmic and Vision Research and were approved by the Experimental Animal Ethics Committee of Xiamen University.

### Subretinal vector injections

The AAV8 (Y733F) was used for packaging the vector DNA as described previously (24). The AAV8 (Y733F)-CBA-hRPE65, AAV8 (Y733F)-CBA-BCL-2-L10 and AAV8(Y733F)-CBA-hRPE65-BCL-2-L10 (10^13^ vector genomes per mL) were used to treat *rd12* mice at postnatal day 14 (P14) respectively. Meanwhile, half-dose AAV8 (Y733F)-CBA-hRPE65 and half-dose AAV8 (Y733F)-CBA-BCL-2-L10 were mixed to treat the mice. After deep anesthesia, subretinal injections were performed as described previously (25). The other eye either remained uninjected. We routinely administered a small amount of fluorescein (0.1 mg/mL final concentration) with the vector to visualize the injection and bleb formation. Such detachments usually resolved within 24 h. After the injection procedure, 1% atropine eye drops and 0.3% tobramycin-dexamethasone eye ointment (Alcon Laboratories) were given three times each day for 3 days. Signs of ocular damage included larger holes in the cornea with accompanying iris-cornea adhesion, hemorrhage in the iris or retina, or damage to the lens, causing cataract formation (26). In animals with minimal apparent surgical complications, only those whose retinal blebs occupied more than half of the retina were retained for further evaluation and we retained at least 6 mice per group for each experiment. The untreated contralateral *rd12* eyes and the age-matched normal C57BL/6 J eyes were used as the control.

### Electroretinography

At 6 months and 12 months posttreatment, retinal function was assessed using the IRC ERG system (Chongqing, China). Briefly, mice were dark-adapted at least 12 h. The animals were anesthetized with inhalation (0.6 L/min) of 5% isoflurane for induction phase and then 1.5% isoflurane for maintenance phase. Pupils were dilated with atropine/phenylephrine under dim red light. Once dilated, animals were exposed to full-field white light flashes at 3.00 cd s/meter^2^ (cd s/m^2^) under scotopic conditions. Responses were averaged and analyzed using the RetiMINNER 4.0 software.

### *In vivo* imaging of mice retinas

Following ERG recording, retinal optical coherence tomography (OCT) imaging was immediately performed using a Small Animal Retinal Imaging System (OptoProbe; OPIMG-L, UK) under anesthesia. We adjusted a multi-directional platform to orient the fully dilated pupil toward a subjective lens for mouse use. One drop of 2.5% hydroxypropyl methylcellulose was administrated to eyes before examination to ensure better OCT imaging, which shows *in vivo* cross-section. Corresponding outer nuclear layer (ONL) thicknesses for untreated, treated *rd12*, and uninjected age-matched C57BL/6 J eyes were compared at the same location, approximately 0.3 mm ventral to the optic papilla.

### Histology

At 12 months posttreatment, following OCT examination, eyes of euthanized mice were enucleated mice and immersed in 4% paraformaldehydes solution and embedded in paraffin. Sections were stained with hematoxylin and eosin. The posterior pole retinas were then examined and photographed with a digital light microscope (Eclipse 50i, Nikon, Tokyo, Japan) and the ONL thickness was measured using Image J (NIH).

### Immunohistochemistry

Eyes from untreated, treated *rd12* mice and uninjected age-matched C57BL/6 J mice, were enucleated and the eyecups were processed as described previously (27). Eyecups were embedded in optimal cutting temperature compound, cut into sagittal sections (10 μm). For immunofluorescence staining, cryosections were permeated with 0.2% Triton X-100. After washing the sections with PBS, sections were blocked in 2% bovine serum albumin (BSA) for 1 h at room temperature, then incubated with anti-RPE65 (1:250, ab13826, Abcam) and anti-Rhodopsin (1:200, A7245, ABclonal) overnight at 4°C. The slides were then washed three times each with PBS and incubated with secondary antibodies for 1 h in the dark at room temperature, followed by three washes each with PBS. Sections were then counterstained with DAPI and securing with a coverslip.

### TUNEL staining

The cell apoptosis was measured by the TMR (red) Tunel Cell Apoptosis Detection Kit (Servicebio, G1502) according to the manufacturer’s instructions. Sections were counterstained with DAPI, mounted and photographed with a laser scanning confocal microscope (FV1000MPE-B, Olympus).

### Western blotting

Neural retinas and adjacent RPE-choroid complexes were dissected from mouse eyeballs as previously reported (28). Protein was extracted with RIPA buffer containing protease inhibitor. Equal amounts of protein extracts were subjected to electrophoresis on 10% SDS-PAGE and then transferred to PVDF membrane. The membranes, 2 h after being blocked with 5% BSA at room temperature, the membranes were incubated with primary antibodies *RPE65* (1:5000, ab13826, Abcam), Rhodopsin (1:1000, ab5417, Abcam) or β-actin (1:5000, AC028, ABclonal) at 4°C overnight, followed by incubation with the corresponding secondary antibodies (1:5000 dilution) for 1 h at room temperature. The results were visualized by enhanced chemiluminescence reagents and recorded with a ChemiDoc XRS+ Imaging system (Bio-Rad).

### Statistical analysis

Statistical analyses were conducted with Prism software (GraphPad, La Jolla, CA). Data were presented as means ± SD. Significant differences between two groups were compared by an independent samples t-test and a one-way ANOVA comparing more than two groups. A *p* value of <0.05 was considered significant.

## Results

### Retinal coverage area following subretinal injections of various vectors

**Figure 1:**
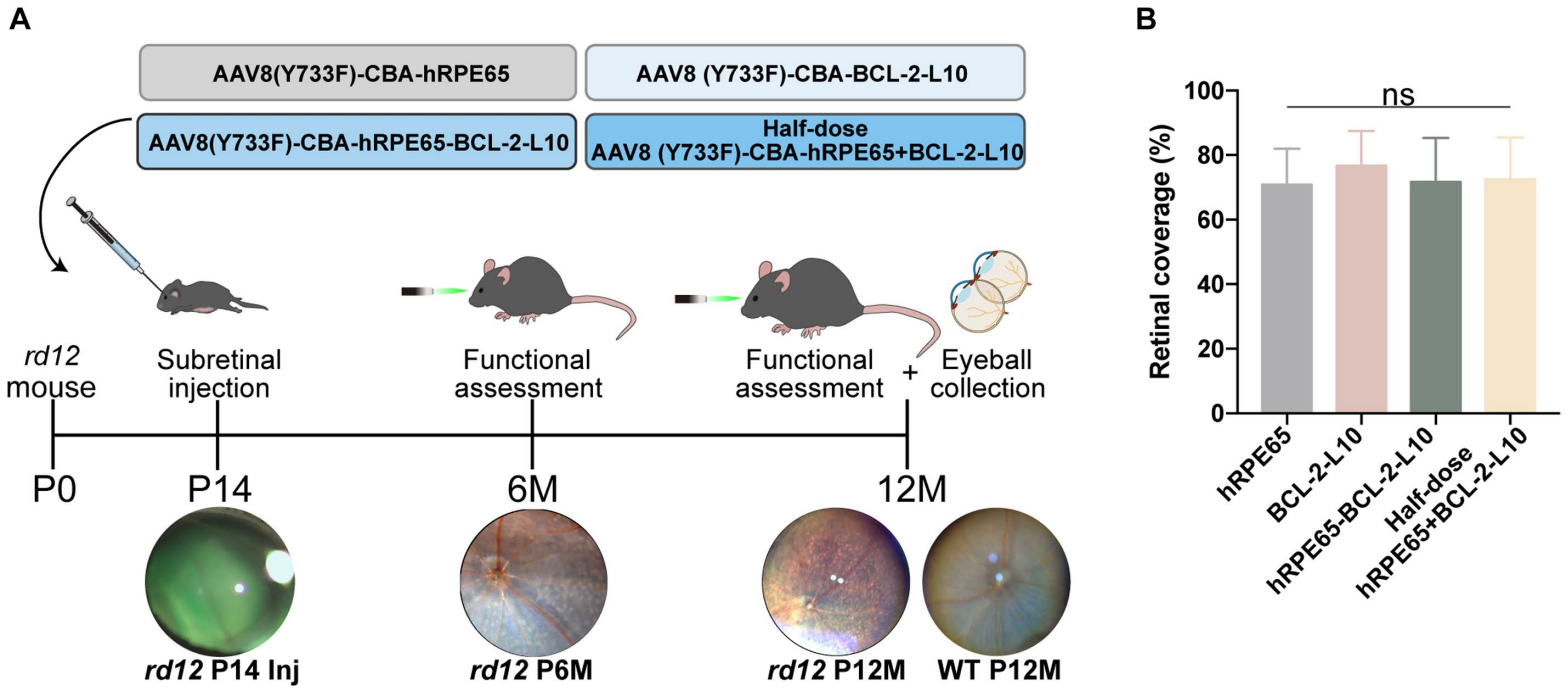
Study design and the extent of the diffusion of subretinally injected vector in *rd12* mouse retinas. **(A)** Experimental timeline to evaluate visual function and histological changes in *rd12* mice after treatment. **(B)** Retinal coverage area was quantified as described previously (29). Bar graphs present the quantification of retinal coverage area depicted as mean ± SD (*n* = 6). ns *p* > 0.05.

To assess the therapeutic effects of the four vector administrations, we initially performed subretinal injection of the vectors at postnatal day 14 (P14) (Figure 1A). Subretinal injection can lead to the separation of the neuroretina and RPE layer, leading to retinal detachment (29, 30). The 0.1% sodium fluorescein (green) was added to the vector solution to improve the identification of the blebs filled, and the diffusion extent was quantified by green blebs. In the treated *rd12* eyes, the retinal blood vessels are distinctly observable on the blebs with green dye underneath, signifying a successful injection (Figure 1A). There was no significant difference (*p* > 0.05) in the area of the retina covered by diffusing vectors between the AAV8(Y733F)-CBA-hRPE65 (hRPE65) group (70.83% ± 4.55%), AAV8(Y733F)-CBA-BCL-2-L10 (BCL-2-L10) group (76.67% ± 4.41%), AAV8 (Y733F)-CBA-hRPE65-BCL-2-L10 (hRPE65-BCL-2-L10) group (71.67% ± 5.58%) and mixture of half-dose AAV8 (Y733F)-CBA-hRPE65 and half-dose BCL-2-L10 (half-dose hRPE65 + BCL-2-L10) group (72.5% ± 5.28%) (Figure 1B).

### Early postnatal AAV-treatment restores ERG photo responses of *rd12* eyes

**Figure 2:**
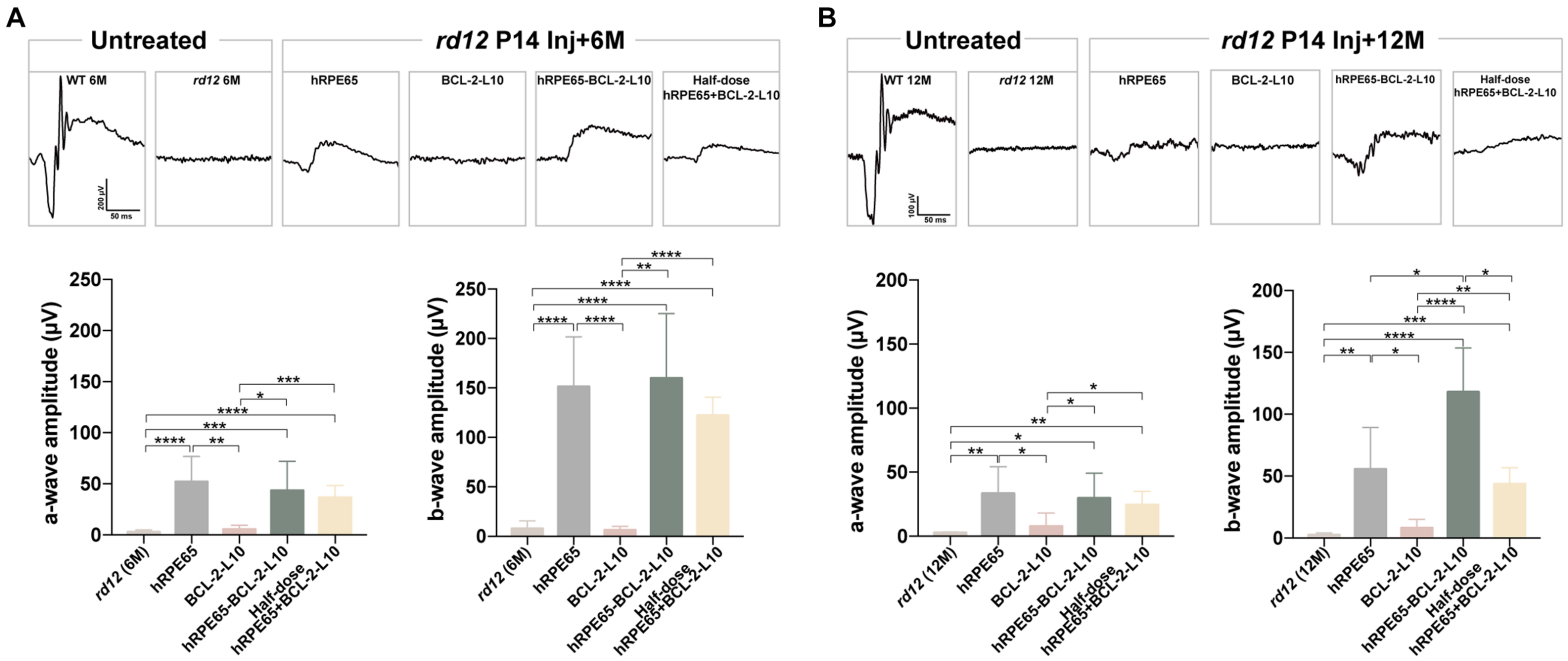
Scotopic ERGs in treated and untreated *rd12* eyes at 6 months and 12 months after P14 treatment. Scotopic ERGs were activated by a bright light with an intensity of 3.00 cd s/m^2^ at 6 months after P14 treatment **(A)** and 12 months after P14 treatment **(B)**. The a- and/or b-wave amplitudes were compared between the treated and control groups (*n* = 4–6). ns *p* > 0.05, *p < 0.05, **p < 0.01, ***p < 0.005, ****p < 0.001.

Scotopic ERGs were conducted on *rd12* mice at both the 6-month and 12-month time points subsequent to treatment with the four distinct vector administrations. Under dark adaptation, the bright stimulus was set at a 3.00 cd s/m^2^ intensity to effectively stimulate both rod and cone responses (31). At 6 months after P14 treatment, rescue of dark-adapted ERG responses was observed and maintained in *rd12* eyes treated with AAV8 (Y733F)-CBA-hRPE65, AAV8 (Y733F)-CBA-hRPE65-BCL-2-L10, mixture of half-dose AAV8(Y733F)-CBA-hRPE65 and half-dose BCL-2-L10 *rd12* eyes, whereas ERG responses were nearly extinguished in untreated 6-month-old *rd12* eyes and *rd12* eyes treated with AAV8 (Y733F)-CBA-BCL-2-L10 (Figure 2A). Among four different AAV-treatments, the ERG amplitudes of the *rd12* eyes treated with AAV8 (Y733F)-CBA-hRPE65-BCL-2-L10 were highest (lower than that of normal uninjected C57BL/6 J mice) followed by the *rd12* eyes treated with AAV8 (Y733F)-CBA-hRPE65 and *rd12* eyes treated with mixture of half-dose AAV8 (Y733F)-CBA-hRPE65 and half-dose BCL-2-L10. Nevertheless, at 12 months after P14 treatment, the ERG amplitudes exhibited a reduction across all groups. Notably, no significant disparity in a-wave or b-wave amplitude was observed between *rd12* mice treated with AAV8 (Y733F)-CBA-hRPE65-BCL-2-L10 and the same mice assessed at 6 months after P14 treatment (Figure 2B).

### Longer-term protective effect of early postnatal AAV-treatment on retinal structure in *rd12* eyes

**Figure 3:**
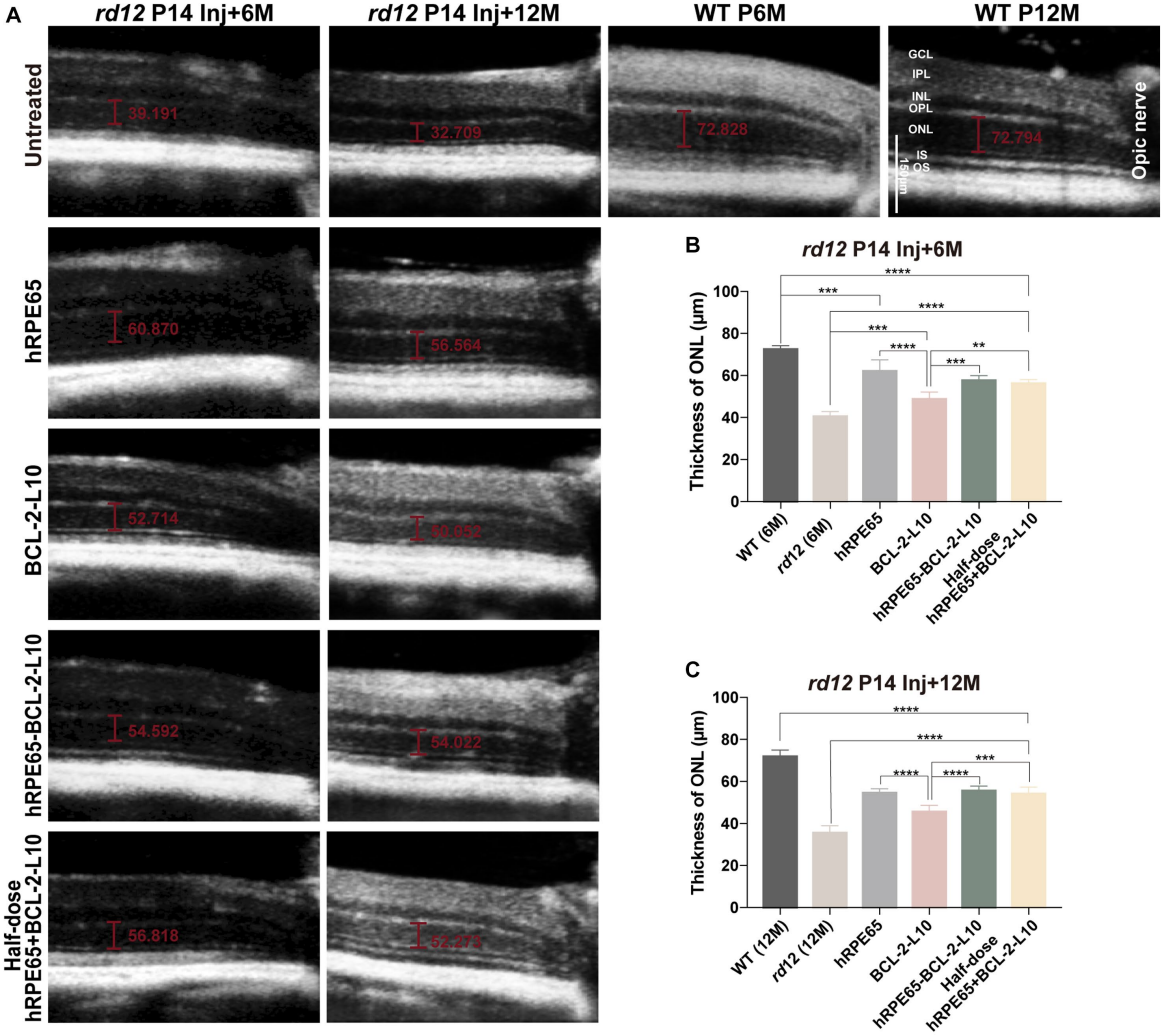
OCT imaging of untreated and treated eyes from *rd12* mice. **(A)** OCT images at the same location were acquired from the untreated and treated eyes from *rd12* mice at 6 months and 12 months after P14 treatment. Scale bars, 150 μm. **(B)** At 6 months after P14 treatment, the thickness of ONL was compared among the different groups (*n* = 4–6). **(C)** At 12 months after P14 treatment, the thickness of ONL was compared among the different groups (*n* = 4–6). **p < 0.01, ***p < 0.005, ****p < 0.001. OS, outer segments, IS, inner segments, ONL, outer nuclear layer, OPL, outer plexiform layer, INL, inner nuclear layer, IPL, inner plexiform layer, GCL, ganglion cell layer.

Research findings have indicated that a reduction in the thickness of outer nuclear layer (ONL) with advancing age in *rd12* mice. Specifically, the ONL of *rd12* mice at 7 months of age exhibited 6 to 7 layers of nuclei, in contrast to the 10 to 11 layers observed in normal C57BL/6 J mice (32). Guided by the results, we focused on investigating longer-term changes in the ONL. The OCT enables the noninvasive evaluation of ONL thickness in eyes subjected to treatment as compared to those that were untreated. In order to compare structural differences, ONL thickness was meticulously measured at a consistent location (0.3 mm temporal to the optic nerve) across all scrutinized eyes (Figure 3A). At 6 months following treatment, significant difference was found in ONL thickness among the untreated and treated *rd12* mice (Figure 3B). The results indicated that although *rd12* mouse eyes treated with AAV8 (Y733F)-CBA-BCL-2-L10 had a thicker ONL structure than the untreated *rd12* eyes (48.64 ± 1.54 μm vs. 40.52 ± 0.82 μm, *p* < 0.05), the other three vector administrations (AAV8 (Y733F)-CBA-hRPE65, AAV8 (Y733F)-CBA-hRPE65-BCL-2-L10, mixture of half-dose AAV8 (Y733F)-CBA-hRPE65 and half-dose BCL-2-L10) demonstrated a more effective therapeutic effect (62.07 ± 2.68 μm, 57.61 ± 0.95 μm and 56.2 ± 0.84 μm). Nonetheless, significant differences remained when compared to WT mice at 6 months of age (72.41 ± 0.79 μm).

According to the ERG findings, a more substantial recovery of visual function was observed in *rd12* mice treated with AAV8 (Y733F)-CBA-hRPE65 and *rd12* mice treated with AAV8 (Y733F)-CBA-hRPE65-BCL-2-L10, in comparison to the other two vector administrations, at 12 months following treatment. Therefore, a comparative analysis was conducted on the alterations in ONL thickness between *rd12* mice treated with AAV8 (Y733F)-CBA-hRPE65 and those treated with AAV8 (Y733F)-CBA-hRPE65-BCL-2-L10, revealing no significant difference (54.55 ± 0.83 μm vs. 55.57 ± 0.77 μm, *p* > 0.05) (Figure 3C). Remarkably, a slight decrease in ONL thickness was observed in *rd12* mice treated with AAV8 (Y733F)-CBA-hRPE65-BCL-2-L10 at 12 months following treatment when compared to *rd12* mice at the 6 months following treatment (55.57 ± 0.77 μm vs. 57.61 ± 0.95 μm, *p* > 0.05). Furthermore, the thickness of the ONL in WT mice at 12 months of age exhibited minimal change compared to that in WT mice at 6 months of age (72.41 ± 0.79 μm vs. 71.89 ± 1.768 μm, *p* > 0.05). Conversely, a significant reduction in ONL thickness was evident in *rd12* mice treated with AAV8 (Y733F)-CBA-hRPE65 (54.55 ± 0.83 μm vs. 62.07 ± 2.68 μm, *p* < 0.05).

**Figure 4:**
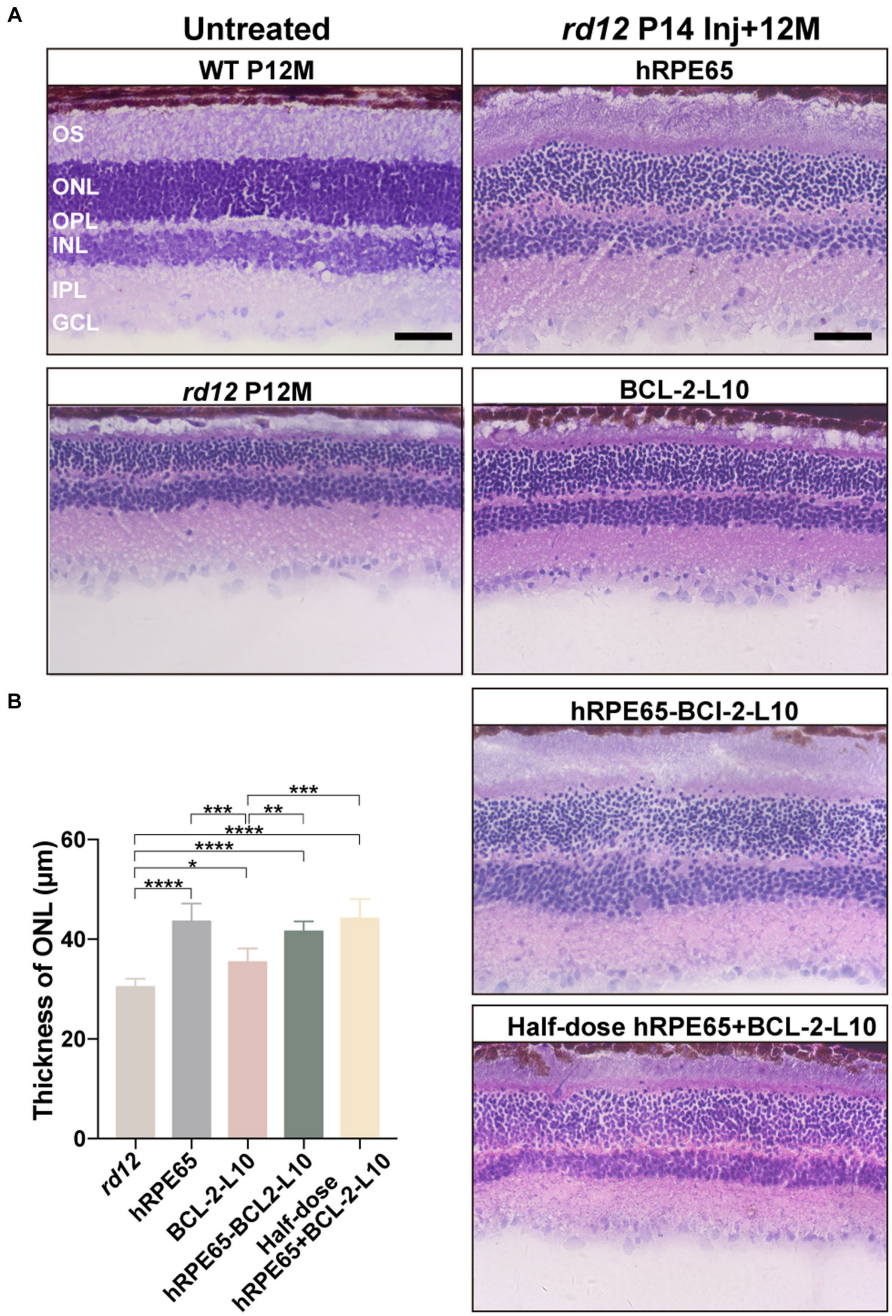
Light microscopic images of untreated and treated retinal sections from *rd12* and normal mice. **(A)** Hematoxylin and eosin-stained retinal cross-sections from a 12-month-old uninjected normal C57BL/6 J eye, untreated and treated eyes from *rd12* mice at 12 months after P14 treatment. Scale bars, 50 μm. **(B)** Light micrographs were taken from the posterior pole segment of the retina. The ONL thickness was compared among the different groups (*n* = 4–6). *p < 0.05, **p < 0.01, ***p < 0.005, ****p < 0.001.

Following OCT examination at 12 months after P14 treatment, *rd12* mice were sacrificed and eyes enucleated for histology (Figure 4A). Higher-magnification images showed that the outer segment (OS) length and ONL thickness became thinner in the untreated *rd12* retinas compared to a normal uninjected C57BL/6 J mice. However, approximately 2/3 of the normal OS length and ONL thickness were maintained in the AAV8 (Y733F)-CBA-hRPE65, AAV8 (Y733F)-CBA-hRPE65-BCL-2-L10, mixture of half-dose AAV8 (Y733F)-CBA-hRPE65 and half-dose BCL-2-L10 treated *rd12* retinas compared to a normal uninjected C57BL/6 J retinas. Significant differences were found in ONL thickness among the untreated and treated *rd12* mice (Figure 4B). In addition, compared to untreated *rd12* retinas, the ONL thickness also became thicker in the AAV8 (Y733F)-CBA-BCL-2-L10 treated *rd12* retinas (30.41 ± 0.68 μm vs. 35.4 ± 1.12, *p* < 0.05), but OS length was not restored. Here, results of retinal morphology were consistent with the *in vivo* OCT measurements.

### Early postnatal dual-gene therapy restores *RPE65* and Rhodopsin expression, delays photoreceptor cell apoptosis in *rd12* eyes

**Figure 5:**
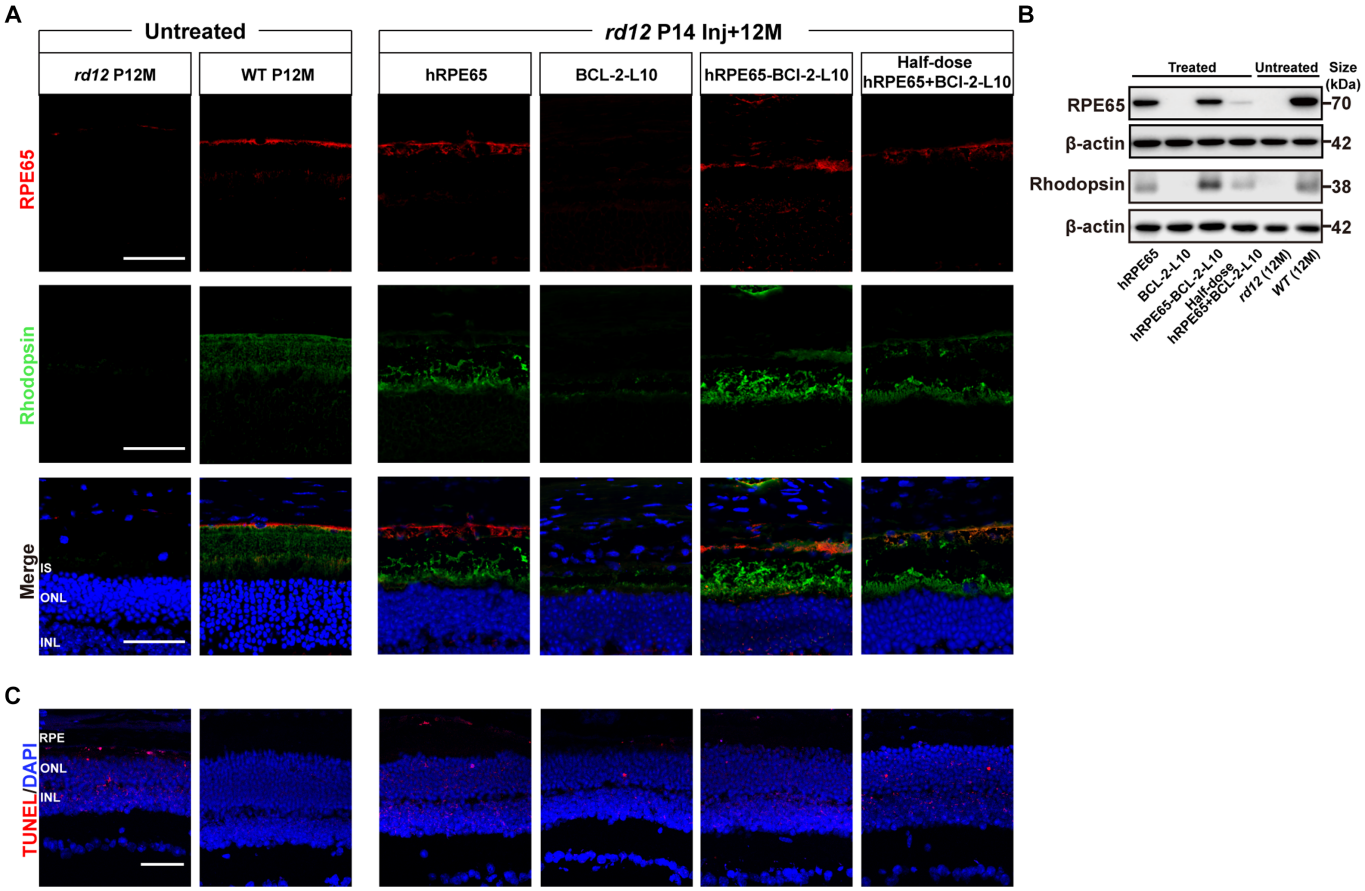
RPE65 and Rhodopsin expression and TUNEL assay in 12-month-old *rd12* mice with treatment. **(A)** RPE65 immunostaining (red) and Rhodopsin immunostaining (green) of representative eye cross sections of treated mice. Nuclei were stained with DAPI (blue). Scale bars, 50 μm. **(B)** Western blot analysis to detect RPE65 (70 kDa) expression in mouse RPE tissue lysate and Rhodopsin (38 kDa) expression in mouse retina tissue lysate 12 months post injection. **(C)** TUNEL assay (red) in treated eyes from *rd12* mice at 12 months after P14 treatment. Nuclei were stained with DAPI (blue). Scale bars, 50 μm.

At 12 months post injection, we assessed the restoration of *RPE65* and Rhodopsin protein in treated eyes through both immunofluorescence and western blotting (Figures 5A,B). In comparison to 12-month-old uninjected *rd12* mice, the expression of *RPE65* was restored in the retinas of three other treated groups, excluding *rd12* mice treated with AAV8(Y733F)-CBA-BCL-2-L10 (Figure 5A). Meanwhile, the expression of Rhodopsin in the retinas of *rd12* mice treated with the four vector administrations exhibited varying degrees of recovery (Figure 5A). Further western blot analysis revealed that the *RPE65* bands were observed in the RPE tissue lysates from the AAV8 (Y733F)-CBA-hRPE65, AAV8 (Y733F)-CBA-hRPE65-BCL-2-L10, mixture of half-dose AAV8 (Y733F)-CBA-hRPE65 and half-dose BCL-2-L10 groups (Figure 5B). In particular, the expression of Rhodopsin in mouse retina tissue lysate of *rd12* mice treated with AAV8 (Y733F)-CBA-hRPE65-BCL-2-L10 demonstrated significant advantages in the four vector administrations.

To evaluate cell apoptosis, we conducted TUNEL staining, which revealed a significant occurrence of photoreceptor cell apoptosis in the RPE and ONL of 12-month-old untreated *rd12* eyes (Figure 5C). At 12 months after p14 injection of AAV8 (Y733F)-CBA-hRPE65, cell apoptosis persisted in both the RPE and ONL in the treated *rd12* eyes. However, the delivery of *Bcl-2L10* gene reduced the photoreceptor cell apoptosis, especially the RPE cells.

## Discussion

*RPE65*-associated LCA comprises around 4% to 16% of reported cases, representing one of the most severe forms of inherited retinal dystrophy (2). Previous reports indicate that mutations in the *RPE65* gene also lead to milder forms of late-onset and progressive rod-cone dystrophy, in contrast to mutations in other genes associated with LCA (33, 34). Depigmentation and whitish deposits outside the macula are noted (35). *rd12* mouse, a naturally occurring LCA2 model with *RPE65* mutation, which displays small, evenly spaced yellowish white dots throughout their retinas (32).

For *RPE65* mutation-associated LCA, alternative therapeutic approaches are available, including exogenous supplementation of 11-cis-retinaldehyde (11cRAL) which serves as the chromophore for retinal photopigments. However, exogenous 11cRAL supplementation only recovered short-term retinal function, exhibiting no discernible benefits 10 days post the final treatment (36). In 2017, a significant milestone was reached with the FDA’s approval of a gene-based drug for commercial use. This achievement marked a substantial breakthrough in the treatment of *RPE65*-deficient LCA2. Amidst the ongoing steady development of gene therapy, the most straightforward strategy involves gene augmentation or replacement. In this strategy, a fully functional copy of a gene is delivered into affected cells in order to restore expression of an inadequately functioning gene (37). Unfortunately, the gradual progression of apoptosis, which persists, is frequently disregarded. Especially, gene augmentation or replacement strategy can temporarily restore some visual function or vision in middle-advanced LCA2 patients, however, most studies at later intervals have shown a lack of durability of the improvements (12–15). In our study, we discovered that the delivery of *RPE65* gene alone led to restoration of certain visual function, aligning with findings from prior research (25). However, after 12 months of long-term observation following the delivery of *RPE65* gene, there was a significant decline in visual function in mice compared to 6 months after the injection. Additionally, the thickness of the ONL of the retina decreased. Results from TUNEL staining revealed that cell apoptosis, especially in RPE cells, continued to occur. The enduring therapeutic efficacy of supplementing the *RPE65* gene is notably diminished, indicating the preservation of both the quantity of visual cells and visual function over an extended period post-treatment proves challenging.

Bcl-2 apoptotic pathway is involved in RPE65-dependent apoptosis of photoreceptors, plays a crucial role in LCA disease (21, 23, 38). Anti-apoptotic therapy constitutes an additional treatment avenue. In this study, we also constructed the AAV8 (Y733F)-CBA-BCL-2-L10 vector and administered subretinal injections to mice at P14. In clinic, thinning of retinal thickness, especially the ONL layer, and loss of lamination in the retina are more prominent in patients older than 30 years of age (35, 39). The ONL appeared thinner in the *rd12* mice at 7 months of age, and the OS were obviously shorter (32). These phenomena were observed in our results through the analysis of OCT and HE staining. Furthermore, the delivery of *Bcl-2L10* gene exhibited a capability to retard the apoptosis of photoreceptor cells. This could, in part, explain the observed augmentation in retinal ONL thickness when contrasting *rd12* mice treated with the AAV8 (Y733F)-CBA-BCL-2-L10 vector to untreated *rd12* mice. However, we observed that *rd12* mice treated with the AAV8 (Y733F)-CBA-BCL-2-L10 vector may not recover the expression of RPE65, ultimately resulting in the inability to recover visual function. Overall, although anti-apoptotic therapy can partially postpone the apoptosis of photoreceptor cells, the approach lacks stability and proves ineffective in certain mice. Furthermore, reinstating visual function remains unattainable due to the persisting fundamental ailment.

A limitation of AAV vectors is their constrained packaging size in comparison to other viral vectors (40). Initially, we mixed a half-dose of AAV8 (Y733F)-CBA-hRPE65 vector and a half-dose AAV8 (Y733F)-CBA-BCL-2-L10 vector for administration via subretinal injection. Subsequently, a comparable therapeutic outcome was observed after a 6-month treatment period, resembling that achieved through a singular injection of AAV8 (Y733F)-CBA-hRPE65 vector. This observation suggests that the co-administration of the two vectors successfully accomplished the objective of reinstating visual function in *rd12* mice and retarding the pace of visual cell apoptosis. However, due to the dosage reduction resulting from the halved dose, a substantial decrease in visual function was observed by 12 months after treatment. Regrettably, the long-term treatment effect was unsatisfactory. Later on, we resolved the challenge of low transfection efficiency associated with using a single AAV vector to concurrently express two proteins, and constructed a dual-gene fragment vector encompassing both *hRPE65* and *Bcl-2L10*. Observations revealed that following a 6-month treatment period, the therapeutic efficacy resembled that achieved through the administration of the AAV8 (Y733F)-CBA-hRPE65 vector alone, as well as through the concurrent administration of the two mixed vectors. Extended observations up to 12 months after treatment revealed that, unlike the other three groups, the thickness of the ONL in the mouse retina had experienced minimal reduction compared to the state at 6 months post-injection. Visual function also exhibited no significant deterioration, although it cannot be fully restored to a normal state. Additionally, at the 12-month post-injection there was a notable reduction in the number of apoptotic RPE cells and ONL cells within the treated *rd12* mice compared to untreated mice. This approach exhibited a lasting effect of up to 12 months post-treatment, showcasing its remarkable long-term efficacy. In the future, we will shift our focus towards optimizing strategies based on the dual-gene vector foundation. This includes conduction a more in-depth exploration of the therapeutic effects of the dual-gene vector at different doses on *rd12* mice retina, while simultaneously concentrating on observing the longer treatment effects.

**Figure 6:**
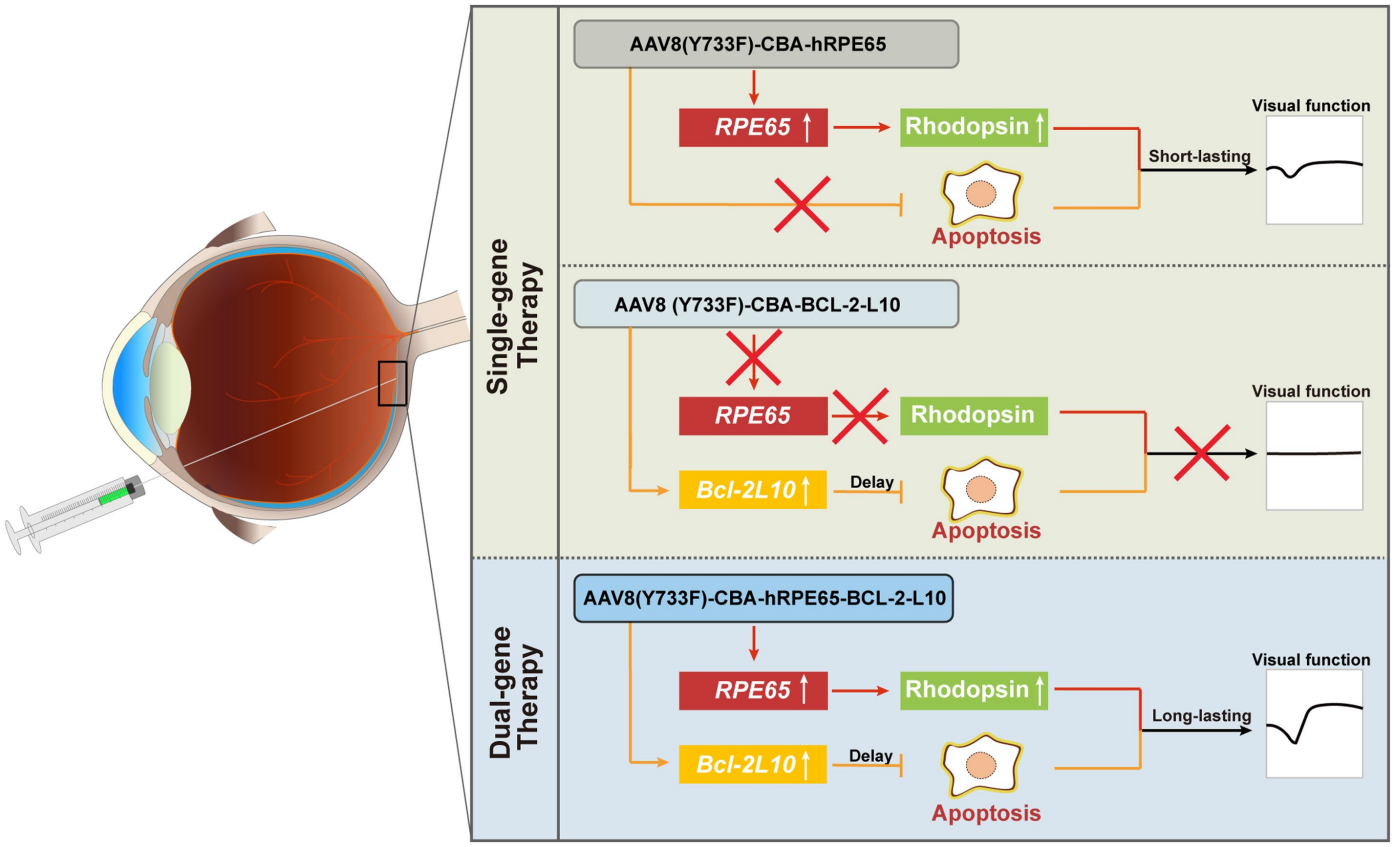
Summary schematic. For dual-gene therapy, on the one hand, the delivery of the *Bcl-2L10* gene can suppress photoreceptor cell apoptosis, consequently prolonging their lifespan and preserving cell quantity. On the other hand, the delivery of the *RPE65* gene enhances Rhodopsin protein expression, resulting in an improvement in photoreceptor cell functionality. By precenting photoreceptor cell apoptosis and restoring visual cell function, a sustained therapeutic effect is achieved.

In conclusion, our findings demonstrate the enduring efficacy of the therapeutic approach that involves inhibiting photoreceptor cell apoptosis while concurrently supplementing *RPE65* (Figure 6). Although the dual-gene therapy strategy is presently in initial phases, our research has the potential to yield valuable insights and offer significant guidance for individuals at intermediate and advanced stages of LCA2.

## Data availability statement

The original contributions presented in the study are included in the article/supplementary material, further inquiries can be directed to the corresponding author.

## Ethics statement

The animal study was approved by Animals in Ophthalmic and Vision Research and were approved by the Experimental Animal Ethics Committee of Xiamen University. The study was conducted in accordance with the local legislation and institutional requirements.

## Author contributions

YL: Data curation, Writing – original draft, Formal analysis. JT: Data curation, Writing – original draft. CY: Formal analysis, Writing – original draft. DXu: Writing – original draft. DXi: Writing – original draft. JP: Funding acquisition, Project administration, Supervision, Validation, Writing – review & editing.
